# Simultaneous Colorimetric Detection of a Variety of *Salmonella* spp. in Food and Environmental Samples by Optical Biosensing Using Oligonucleotide-Gold Nanoparticles

**DOI:** 10.3389/fmicb.2019.01138

**Published:** 2019-05-31

**Authors:** Irwin A. Quintela, Benildo G. de los Reyes, Chih-Sheng Lin, Vivian C. H. Wu

**Affiliations:** ^1^Produce Safety and Microbiology Research Unit, U.S. Department of Agriculture, Agricultural Research Services, Western Regional Research Center, Albany, CA, United States; ^2^School of Food and Agriculture, University of Maine, Orono, ME, United States; ^3^Department of Plant and Soil Science, Texas Tech University, Lubbock, TX, United States; ^4^Department of Biological Science and Technology, National Chiao Tung University, Hsinchu, Taiwan

**Keywords:** gold nanoparticles, optical biosensor, *ttrRSBCA*, colorimetric, oligonucleotides, *Salmonella*

## Abstract

Optical biosensors for rapid detection of significant foodborne pathogens are steadily gaining popularity due to its simplicity and sensitivity. While nanomaterials such as gold nanoparticles (AuNPs) are commonly used as signal amplifiers for optical biosensors, AuNPs can also be utilized as a robust biosensing platform. Many reported optical biosensors were designed for individual pathogen detection in a single assay and have high detection limit (DL). *Salmonella* spp. is one of the major causative agents of foodborne sickness, hospitalization and deaths. Unfortunately, there are around 2,000 serotypes of *Salmonella* worldwide, and rapid and simultaneous detection of multiple strains in a single assay is lacking. In this study, a comprehensive and highly sensitive simultaneous colorimetric detection of nineteen (19) environmental and outbreak *Salmonella* spp. strains was achieved by a novel optical biosensing platform using oligonucleotide-functionalized AuNPs. A pair of newly designed single stranded oligonucleotides (30-mer) was displayed onto the surface of AuNPs (13 nm) as detection probes to hybridize with a conserved genomic region (192-bases) of *ttrRSBCA* found on a broad range of *Salmonella* spp. strains. The sandwich hybridization (30 min, 55°C) resulted in a structural formation of highly stable oligonucleotide/AuNPs-DNA complexes which remained undisturbed even after subjecting to an increased salt concentration (2 M, final), thus allowing a direct discrimination via color change of target (red color) from non-target (purplish-blue color) reaction mixtures by direct observation using the naked eye. In food matrices (blueberries and chicken meat), nineteen different *Salmonella* spp. strains were concentrated using immunomagnetic separation and then simultaneously detected in a 96-well microplate by oligonucleotide-functionalized AuNPs after DNA preparation. Successful oligonucleotide/AuNPs-DNA hybridization was confirmed by gel electrophoresis while AuNPs aggregation in non-target and control reaction mixtures was verified by both spectrophotometric analysis and TEM images. Results showed that the optical AuNP biosensing platform can simultaneously screen nineteen (19) viable *Salmonella* spp. strains tested with 100% specificity and a superior detection limit of <10 CFU/mL or g for both pure culture and complex matrices setups. The highly sensitive colorimetric detection system can significantly improve the screening and detection of viable *Salmonella* spp. strains present in complex food and environmental matrices, therefore reducing the risks of contamination and incidence of foodborne diseases.

## Introduction

Nanomaterials are commonly incorporated in the development of optical biosensors to enhance its sensitivity (Pérez-López and Merkoçi, 2011). Nanomaterials such as gold nanoparticles (AuNPs), gold nanorods (GNRs), and quantum dots (QDs) are also utilized to increase capture efficiency of analytes, signal amplification and improvement of detection limit (DL). AuNPs within 13–20 nm diameter range possess excellent dispersity and biocompatibility with relative ease of functionalization and integration into colorimetric, electrochemical, scattering and fluorescence detection methods targeting bacterial cells, nucleic acids, toxins, viruses and other analytes of interests (Yola et al., 2013, 2015b; Chen et al., 2014; Quintela et al., 2015). After modification, functionalization or physico-chemical enhancement, the resulting AuNPs ultimately lead to usable morphological transformations such as color change via dispersion and aggregation (Chen et al., 2014).

AuNPs surface modification via ligand exchange is an excellent approach in the design and development of biosensors (Zhu et al., 2017). For various biosensing purposes, AuNPs have also facilitated enhancement of electrode conductivity and increased sensitivity (Yola and Atar, 2014). Successful detection of DNA hybridization using an impedimetric biosensor integrated with AuNPs has been reported (Gupta et al., 2013). In addition, a molecular imprinted electrochemical biosensor based on polyoxometalate and graphene oxide decorated-AuNPs has successfully detected chemicals such as triclosan (TCS) from environmental water samples (Yola et al., 2015b). For colorimetric assay, the bio functionality of AuNPs has been demonstrated in previous works such as by using quaternary ammonium ligands and β-galactosidase enzyme with cationic AuNPs in which its binding event with the target bacterium, *Escherichia coli* XL1, delivered a colorimetric read-out for sensing and quantification (Miranda et al., 2011). Similarly, cationic AuNPs have been non-covalently conjugated with anionic polyparaphenyleneethynylene (PPE) polymers for rapid and efficient bacterial identification in a microarray setup (Phillips et al., 2008). This sensor array relies on the displacement of the fluorescent PPE from the AuNPs quenchers after binding with the target bacteria. Various shapes (i.e., oval and popcorn-shaped) of AuNPs can also be tailored and functionalized for rapid colorimetric detection of significant pathogens (Khan et al., 2011; Afonso et al., 2013). An optical biosensor for *Chlamydia trachomatis* DNA detection that utilized GNRs as molecular probes has been previously reported (Parab et al., 2010). The principle behind the DNA detection is based on the simultaneous biorecognition-mediated hybridization of target DNA in a sandwich type manner with two different capture GNRs-DNA probe which leads to aggregation (Ko and Grant, 2006). It successfully detected PCR amplified *C. trachomatis* pathogen gene (concentration range, 0.25–20 nM). A fiber optic portable biosensor utilizing the principle of fluorescence resonance energy transfer (FRET) was developed for *Salmonella* Typhimurium detection in pork samples and had a detection limit of 10^5^ colony forming unit or CFU/g (Ko and Grant, 2006). Though various molecular and immunological-based optical biosensors have been widely studied and designed for rapid detection and identification of foodborne bacterial pathogens, many of these methods are only capable of detecting a single pathogen with high detection limit (DL) approximately 10^4^–10^5^ CFU/mL. An aptamer-based colorimetric detection assay by Bayraç et al. (2017) was able to specifically screen the target strain, *S.* Enteriditis, however, when it was applied on real food sample (i.e., milk), the detection limit was only 10^3^ CFU/mL. A colorimetric aptasensor-based platform based on the peroxidase-like activity of ZnFe_2_O_4_-reduced graphene oxide nanostructures with a sensitivity of 2 log CFU/mL (milk sample) has been reported by Wu et al. (2017); however, it was designed only for single pathogen (*S*. Typhimurium) detection. More so, an interesting paper was reported by Abdelhaseib et al. (2016) on BARDOT (bacterial rapid detection using optical scattering technology) that could facilitate the detection of *S*. Enteritidis and *S*. Typhimurium serovars using fiber optic sensor but with a detection limit of 10^3^ CFU/mL from selectively enriched poultry products. Therefore, a more comprehensive system that can detect multiple target pathogens simultaneously in a single assay with superior DL is desired.

*Salmonella* spp. is a significant bacterial foodborne pathogen causing outbreaks and sporadic cases of gastroenteritis (Franklin et al., 2011). The nature and severity of diseases caused by *Salmonella* spp. is based on serotype, host species and presence of virulence factors such as *Salmonella* pathogenicity islands (SPIs), plasmids, toxins, fimbriae, and flagella (Zou et al., 2010). SPIs are small chromosomal regions that carry multiple *Salmonella* spp. virulence genes. *ttrRSBCA* is a locus near SPI-2 that encodes for tetrathionate reductase enzyme for anaerobic respiration. Specifically, *Salmonella* spp. utilizes a sulfur compound tetrathionate which acts as one of the terminal electron acceptors during anaerobic respiration (James et al., 2013).

In this work, we developed a novel approach for simultaneous optical detection of various *Salmonella* spp. strains in contaminated complex matrices (i.e., food and environmental samples) by utilizing oligonucleotide-functionalized AuNPs as a sensitive optical biosensing platform in combination with an efficient sample pooling and immunomagnetic separation (IMS) system that ensure detection of viable cells. It features a highly sensitive detection technology that specifically targets conserved region of *ttrRSBCA* locus by a sandwich hybridization approach. The development of the detection technology that simultaneously and directly detects a comprehensive outbreak bacterial strains in complex matrices can efficiently reduce the risks and incidence of foodborne diseases.

## Materials and Methods

### Reagents and Materials

Gold (III) chloride trihydrate (HAuCl_4_⋅3H_2_O) and sodium citrate (C_6_H_5_Na_3_O_7_) were purchased from Sigma-Aldrich (St. Louis, MO, United States). Sodium chloride (NaCl) and disodium hydrogen phosphate (Na_2_HPO_4_) were obtained from Fisher Scientific (Lawn Fair, NJ, United States). Culture media: Brain Heart Infusion (BHI) broth and Hektoen Enteric (HE) Agar, and MacConkey Agar with Sorbitol (SMAC) were purchased from Neogen (Lansing, MI, United States) while Buffered Peptone Water (BPW) was supplied by Becton, Dickinson and Company (Franklin Lakes, NJ, United States). Pathatrix 5 Pooling *Salmonella* spp. was purchased from Life Technologies (Grand Island, NY, United States) and DNeasy Blood & Tissue Kit for DNA extraction was obtained from Qiagen (Hilden, Germany). Asymmetric PCR (asPCR) primers were customized by Integrated DNA Technologies (Coralville, IA, United States): For (For-192-Sal) 5′-ACC CAC GCG TTT CAT CGG TT-3′ and Rev (Rev-192-Sal) 5′-GCC GGC AAT CCC TAT CAC CC-3′. Two sets of thiol-modified oligonucleotides labeled as Probe 1 and Probe 2 with sequences (P1-Sal 5′-AGC AAC TGG CGG GAG AAA GCG GTC TTG ACG-3) and (P2-192-Sal HS-(CH_2_)_6_ -5′-GCA GGA ACA CCC GAT TGA CTC GTC CGT CCC-3′), respectively, were designed from *S.* Typhimurium complete genome sequence (Gen Bank accession no. CP019649.1), synthesized and thiol-modified by Eurogentec (San Diego, CA, United States). All reagents used in this study were analytical grade and utilized without further treatment. Water was purified (>18.3 MΩ/cm) using a Barnstead Nanopure filtration system (Lake Balboa, CA, United States).

### Bacterial Cultures and Genomic DNA Preparation

All bacterial strains used are listed in Table 1. All bacterial cultures were initially stored in cryogenic beads with Brucella broth and glycerol (CryoSavers; Hardy Diagnostics, Santa Maria, CA, United States) at -80°C before activating them overnight using BHI (Neogen) at 37°C. HE Agar (Neogen) and MacConkey Agar with Sorbitol (Neogen) were used for viable *Salmonella* spp. and STEC O157:H7 ATCC 35150 cell counts, respectively. Genomic bacterial DNA from all samples were extracted using DNeasy Blood & Tissue Kit (Qiagen) following manufacturer’s protocol. Asymmetric PCR (asPCR) was conducted with a highly specific pair of primers, For-192-Sal and Rev-192-Sal (10:1 ratio). All products were kept at -20°C until further use.

**Table 1:** List of bacterial strains, target locus and source.

Code	Bacterial strains	*ttrRSBCA* locus	Source
S	*Salmonella enterica* Agona	+	US FDA
1	*Salmonella enterica* Anatum	+	US FDA
2	*Salmonella enterica* Berta	+	US FDA
3	*Salmonella enterica* Derby	+	US FDA
4	*Salmonella enterica* Dublin	+	US FDA
5	*Salmonella enterica* Enteriditis	+	US FDA
6	*Salmonella enterica* Gallinarum	+	US FDA
7	*Salmonella enterica* Heidelberg	+	US FDA
8	*Salmonella enterica* Infantis	+	US FDA
9	*Salmonella enterica* Javiana	+	US FDA
10	*Salmonella enterica* Kentucy	+	US FDA
11	*Salmonella enterica* Mbandaka	+	US FDA
12	*Salmonella enterica* Montevideo	+	US FDA
13	*Salmonella enterica* Muenster	+	US FDA
14	*Salmonella enterica* Newport	+	US FDA
15	*Salmonella enterica* Oranienburg	+	US FDA
16	*Salmonella enterica* Saintpaul	+	US FDA
17	*Salmonella enterica* Senftenberg	+	US FDA
18	*Salmonella enterica* Thompson	+	US FDA
19	*Salmonella enterica* Typhimurium 14028	+	ATCC
NT	*Escherichia coli* O157:H7 35150	-	ATCC

*US FDA, United States Food and Drug Administration in Silver Spring, MD, United States; ATCC, American Type Culture Collection.*

### Designing and Synthesis of AuNPs-Oligonucleotide Probes

**SCHEME 1:**
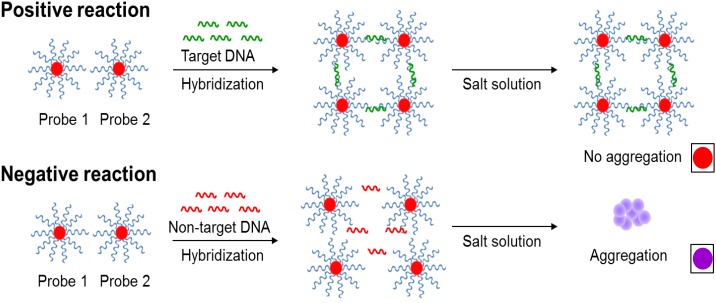
The novel principles behind AuNPs optical biosensing and the schematic representation of DNA sandwich hybridization targeting *ttrRSBCA* locus of *Salmonella* spp. The high complementarity between the AuNPs-oligonucleotide probes (Probes 1 and 2) and *ttrRSBCA* regions allows the occurrence of DNA sandwich hybridization. Probe-target complexes form with high specificity and retain in the solution even after increasing its salt concentration. Positive reaction (*ttrRSBCA* locus) shows no AuNPs-oligonucleotide probes aggregation thus no color change (original red) is observed. Formation of probe-target complexes did not occur in blank or non-target DNA samples; therefore, the salt-induced AuNPs-oligonucleotide probes aggregation occurs that eventually changes the color of the reaction mixtures (from the original red to purplish blue). Color differentiation facilitates direct detection of positive and negative samples by observation using the naked eye. Size not to scale (reproduced with some modifications and with permissions from Quintela et al. (2015); copyright 2015, Royal Society of Chemistry).

AuNPs were synthesized by using citrate reduction method as previously described (Lin et al., 2008). A microplate reader spectrophotometer (BioTek Power Wave XS, Winoskii, VT, United States) confirmed the average size of the newly synthesized AuNPs at 400–700 nm based on wavelength absorption spectra. Probes 1 and 2 (30-mer, 20 μM, 20 μL) were conjugated onto the surface of AuNPs (Supplementary Figure S1). In brief, each oligonucleotide probe was added to AuNPs solution (20 μM, 980 μL) and then subjected to increasing salt concentration (0.05–1.0 M NaCl in 10 mM Na_2_HPO_4_, pH 7.4) for 24 h in water bath at 37°C to maximize the oligonucleotide loading capacity. After incubation, unbound probes were discarded by centrifugation (19,530 ×*g*) for 30 min and the oily-like AuNPs which remained at the bottom of the tube was resuspended in salt buffer solution (NaCl in 10 mM Na_2_HPO_4_, pH 7.4). These freshly synthesized AuNPs-oligonucleotide probes were placed in dark storage at room temperature until further use. Shift in the absorbance peak of AuNPs was measured to confirm successful conjugation of oligonucleotide probes. The novel principles behind the simultaneous AuNPs optical biosensing and the schematic representation of DNA sandwich hybridization targeting *ttrRSBCA* locus which is found in a broad range of *Salmonella* spp. strains is presented in Scheme 1.

### AuNPs Optical Biosensing Assay on Pure Bacterial Cultures

In brief, asPCR products (50 μL) or blank was mixed with AuNPs-oligonucleotide probes (Probe 1 and Probe 2, 50 μL each) and incubated for 30 min at 55°C for DNA sandwich hybridization. All mixtures were then transferred to a 96-well microplate for color challenge test by adding a salt solution (final concentration, 2 M NaCl in 10 mM Na_2_HPO_4_, pH 7.4). The color changes in various reaction mixtures were simultaneously observed within 5 min. The rest of reaction mixtures retained its original red color. Pre- and post-color challenge test absorbance (400–700 nm) and ratio at two wavelengths, 625 nm and 525 nm (A_625/525__nm_), were also collected for spectrophotometric analysis. Gel electrophoresis (3% agarose gel and ethidium bromide) was performed to all pre-color challenge test reaction mixtures (50 μL) to verify the occurrence of a successful oligonucleotide/AuNP-DNA sandwich hybridization.

### Sample Pooling and Detection of Live *Salmonella* spp. Strains in Complex Matrices

**SCHEME 2:**
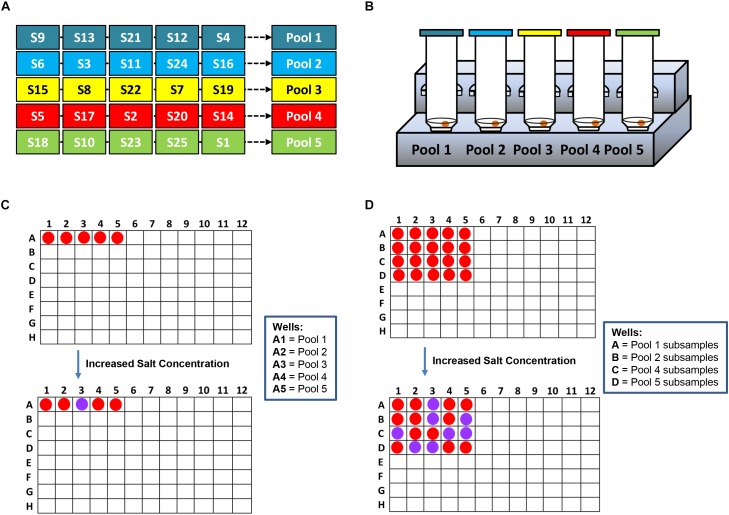
A workflow showing rapid *Salmonella* spp. strains detection in complex matrices using the novel AuNPs optical biosensor coupled with a robust sample pooling plan and an IMS system. **(A)** Twenty-five different individual enriched food or environmental samples are randomly pooled into five groups: Pools 1–5. **(B)** Each pool is circulated in the IMS system for capture and concentration of *Salmonella* spp. strains. Genomic DNA are extracted from the concentrated IMS samples and amplified **(C)** DNA sandwich hybridization reaction mixtures are transfered into a 96-well microplate for color challenge test by increasing its salt concentration. If pools change from red to purplish blue after adding salt, reactions are interpreted as negative for *ttRSBCA*; all of the individual subsamples are therefore *ttrRSBCA-*negative. If pools remain red after adding salt, reaction mixtures are interpreted as positive for *ttrRSBCA* and all of the individual subsamples are further tested. **(D)** AuNPs optical biosensing is conducted for all individual subsamples belonging to the *ttrRSBCA*-positive pools. S = Subsamples.

A robust sample pooling plan was designed to efficiently test post-enriched samples prior to the application of the IMS system, Pathatrix (Matrix MicroScience, Newmarket, United Kingdom) with Pathatrix 5 Pooling *Salmonella* spp. (Life Technologies). In Scheme 2, a workflow shows the rapid simultaneous detection of *Salmonella* spp. strains in complex matrices using the novel AuNPs optical biosensor. In brief, chicken meat with skin was purchased from a local retailer while unwashed frozen blueberries were supplied by a local farmer. Individual overnight cultures of *Salmonella* spp. as listed in Section “Bacterial Cultures and Genomic DNA Preparation” were randomly spiked at 0–9 bacterial cells/g (<10 CFU/g) inoculum levels to food samples (25 g × 25 samples) placed in individual stomacher bags (Fisher Scientific). After which, 225 mL of BPW (Becton, Dickinson and Company) was added, as recommended in the USDA/Food Safety and Inspection Service (FSIS) Microbiology Laboratory Guidebook (MLG) 4.08 for the isolation and identification of *Salmonella* spp. with minor modifications. Samples were homogenized using a Pulsifier (Microbiology International, Frederick, MD, United States) and then enriched for 6 h at 37°C.

Five individual (50 mL each) samples of each kind, either chicken meat with skin or blueberry samples were pooled together. Pooled samples were concentrated (37°C, 30 min) following the manufacturer’s instructions (Pathatrix, Matrix Microscience). For environmental samples, five different sampling sites were randomly chosen for soil with chicken manures (25 g × 5 samples) samples collection. Genomic DNA was extracted as described in section “Bacterial Cultures and Genomic DNA Preparation”. Detection was conducted by applying the previous protocols used for AuNPs optical biosensing of pure cultures. AuNP optical biosensing was performed in parallel with selective plating (HE Agar) for direct comparison.

### Transmission Electron Microscopy (TEM)

The morphological properties and behavior of AuNPs were analyzed using Philips CM10 TEM (Philips, Eindhoven, Netherlands) operating at 80 kV. Two microliters each of modified AuNPs and reaction mixtures were deposited directly onto 200 mesh carbon-coated copper grids then air-dried for 30–45 min at ambient temperature and pressure for TEM imaging.

### Statistical Analysis

Three trials were performed per each experiment. To evaluate the reproducibility of the assay, all samples tested were in duplicates, and the mean of the data ± standard deviation (S.D.) was analyzed by JMP software using one-way ANOVA for significance (*p* < 0.05). Fisher’s least significance difference (LSD) was used for *post hoc* analysis to confirm the significant differences between groups.

## Results and Discussion

### Functionalization of AuNPs With Single-Stranded Thiolated-Oligonucleotides

AuNPs synthesis using citrate reduction method was based on HAuCl_4_ reduction and instantaneous stabilization in trisodium citrate. To limit the negative effects of ionic interactions of synthesized AuNPs, low concentration of reactants (25 mL, 2.5 mM CHLOROAURIC ACID, 3 ML 38.3 MM sodium citrate solution) was used before resuspending the final product in aqueous salt solution (Gittins and Caruso, 2001). The solution shifted from the initial bluish-gray color to purplish until it completely turned to ruby red solution which was one of the primary attributes of spherical AuNPs within 5–15 nm diameter range (Wuithschick et al., 2015). The controlled and morphologically consistent size of colloidal AuNPs has a direct correlation with the ratio of citrate to gold (Hinterwirth et al., 2013). Generally, AuNPs (≤20 nm diameter, 𝜀 = 10^8^/cm M) in aqueous media has 520 nm plasmon band on the average which makes it very compatible for color differentiation-based optical biosensing (Chen et al., 2014).

To fully take advantage of the useful properties of nanomaterials, its functionalization is necessary which can also preserve its properties and biocompatibilities (DeLong et al., 2010). In this study, we designed highly specific oligonucleotides and conjugated it onto the surface of AuNPs via thiol linkage, HS-(CH_2_)_6_, which was initially introduced chemically to either 5′- or 3′- end of the oligonucleotide probes. The stability of AuNPs-probes is supported by a strong covalent bond that forms between thiol and gold (Au-S) and mediated by the sulfhydryl (SH) functional group (Li et al., 2013). Sulfhydryl compounds are small molecules that exert less steric hindrance which provide thiolated-oligonucleotide with superior accessibility to AuNPs by citrate ions replacement without altering its biological functions (Chegel et al., 2011). Specifically, functionalization of AuNPs involved “salt aging” method which requires excess amount of thiolated-DNA with periodic addition of salt solution until the final salt concentration and salt-stable nanoparticles are achieved. Herein, the final salt concentration in phosphate buffer was 1 M NaCl in 10 mM Na_2_HPO_4_, pH 7.4. Compared to other buffer solutions such as Tris, phosphate buffer has relatively higher DNA loading capacity which makes it suitable for AuNPs functionalization. Previous studies have shown that negatively charged DNA bind to positively charged amine groups that were present in Tris resulting to bigger sizes and eventually reducing the available space (approx. 16% less strands per particle at 1 M NaCl) as well as surface loading capacity of AuNPs (Hurst et al., 2006). It was noteworthy that there was no obvious color change between the pre- and post-AuNPs functionalized solutions. Ultimately, the absorbance peak of AuNPs shifted by 2–3 nm after immobilizing thiolated-oligonucleotide mainly due to increase in its refractive index which was a direct evidence of successful AuNPs surface modification (Supplementary Figure S2).

### AuNPs Optical Biosensing of *Salmonella* spp. Strains in Pure Cultures

In this study, amplified *ttrRSBCA* of *Salmonella* spp. strains from inoculum levels 10–10^3^ and 10^8^ CFU/mL were tested along with STEC O157:H7 ATCC 35150 as non-target and nuclease-free water as blank. It was simultaneously observed by naked eye that the reaction mixture wells of various *Salmonella* spp. strains remained red in color whereas the wells containing STEC O157:H7 ATCC 35150 or blank changed to purplish-blue color after the addition of salt solution (2M, final concentration).

**FIGURE 1:**
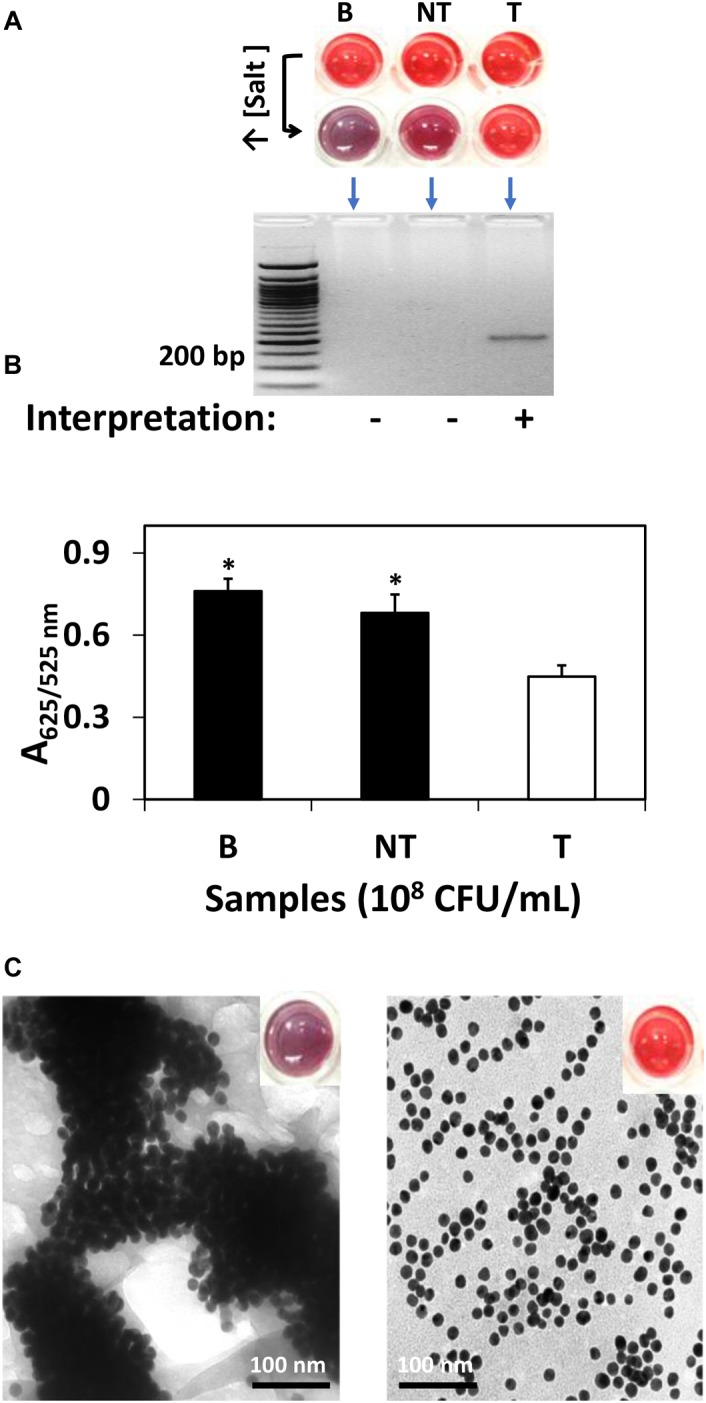
AuNPs optical biosensing targeting *ttrRSBCA* region of *S.* Agona in pure culture setup. **(A)** AuNPs optical biosensing showing three reaction mixtures before and after adding salt solution (50 μL, 2 M NaCl–Na_2_HPO_4_ in final reaction). Both B and NT samples changed from red to bluish-purple color while no color change was observed in T reaction mixture. DNA sandwich hybridization between *ttrRSBCA* and AuNPs-probes was confirmed by a gel band (192-bp). **(B)** A_625/525nm_ of the reaction mixtures after increasing its salt concentration showed significant differences at *p* < 0.05 between B, NT and T **(C)** TEM images confirmed salt induced AuNPs-probe aggregation in B or NT (left image with a bluish-purple solution as inserted) sample while no significant AuNPs aggregation was observed in *S*. Agona (right image with a red color solution as inserted). Both NT and T had 10^8^ CFU/mL bacterial concentration. B = Blank, nuclease-free water; NT = non-target, STEC O157:H7 ATCC 35150; T = target, *S.* Agona. ^∗^Significant difference at *p* < 0.05.

For research purpose, further confirmation was conducted by gel electrophoresis and spectrophotometric analysis. Specifically, as shown in Figure 1, *S*. Agona was selected as the representative strain for AuNPs optical biosensing at 10^8^ CFU/mL inoculum level. In Figure 1A, the successful hybridization of oligonucleotide/AuNPs probes with its complementary region was observed as it retained its original red color after adding salt solution. This can be attributed to the formation of a network and complexes through DNA sandwich hybridization that stabilized AuNPs even in an increased salt concentration. The phosphate groups on the DNA backbone promote interparticle electrostatic repulsion which counteracted the elevated ionic strength of the salt solution as well as the van der Waals attraction force. The interactions between the complimentary probes and target regions may have led to a stronger ordered B-form structure, thus limiting co-localization of AuNPs and allowing the retention of original red color. Successful DNA sandwich hybridization between oligonucleotide/AuNPs and the *ttrRSBCA* of *S*. Agona was also confirmed by the formation of a gel band around the 200-bp region while non-target (STEC O157:H7 ATCC 35150) and blank (nuclease-free water) did not show any bands. The non-target and blank reaction mixtures shifted from red to purplish-blue color primarily due to absence of complementary strands that could have facilitated the network formation and rendered interparticle stability. The repulsion force exerted by AuNPs was weakened and neutralized by the sheer ionic strength of the salt solution that ultimately led to the aggregation of AuNPs.

As an aggregation parameter of AuNPs, empirical measurement was derived to quantify the variation of integrated absorbance of samples after adding salt solution through its absorbance ratio at 625 and 525 nm (A_625/525nm_) wavelengths. This was determined based on the average absorbance of target reaction mixtures which peaked at around 525 nm while blank and non-target reaction mixtures had both absorbance peak at a longer wavelength at 625 nm. The A_625/525nm_ of *S*. Agona after adding salt solution in Figure 1B clearly showed a significant difference at *p* < 0.05 as compared to blank and non-target samples. TEM images in Figure 1C confirmed salt induced AuNPs-probe aggregation in blank or non-target sample while no significant aggregation of AuNPs was observed in *S*. Agona reaction mixture.

**FIGURE 2:**
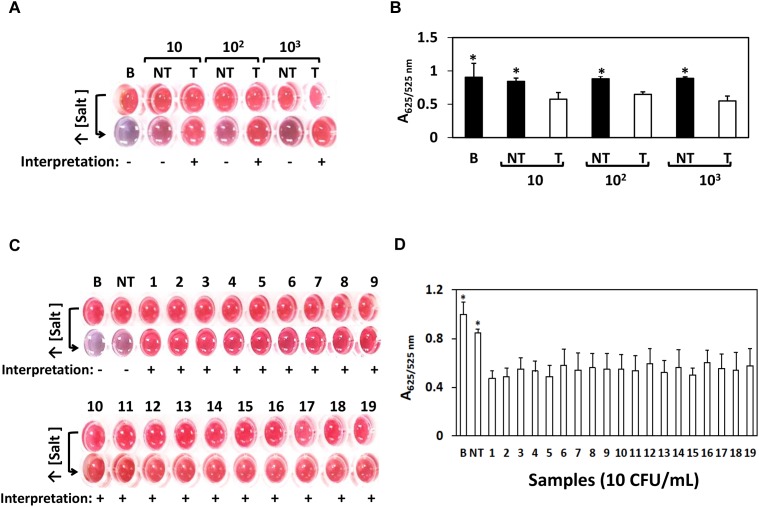
AuNPs optical biosensing – pure culture setup using various *Salmonella* spp. strains. **(A)** AuNPs optical biosensing a 10, 10^2^ and 10^3^ CFU/mL bacterial count levels, showing positive results for T and negative for both B and NT reaction mixtures after adding salt **(B)** A_625/525nm_ of the reaction mixtures after increasing its salt concentration showing significant differences at *p* < 0.05 between B, NT and T **(C)** AuNPs optical biosensing of various *Salmonella* spp. strains at 10 CFU/mL bacterial counts. Reaction mixtures containing B and NT changed from red to purplish blue after adding salt solution while the original color of all target numbered samples remained unchanged. **(D)** A_625/525nm_ of the reaction mixtures presented in panel **(C)** after increasing its salt concentration showing significant differences at *p* < 0.05 between B, NT and T. B = Blank, nuclease-free water; NT = non-target, STEC O157:H7 ATCC 35150, T = target, *Salmonella* spp. strains as numbered 1–19 in Table 1. ^∗^Significant difference at *p* < 0.05.

We next challenged the sensitivity of the AuNPs optical biosensing on various *Salmonella* spp. strains as shown Figure 2. At bacterial levels 10, 10^2^, and 10^3^ CFU/mL, *S.* Typhimurium ATCC 14028 showed positive results while negative for both bank (nuclease-free water) and non-target STEC O157:H7 ATCC 35150 as presented in Figure 2A. The absorbance (A_625/525nm_) in Figure 2B showed significant differences at *p* < 0.05 between target, blank and non-target. Similar results shown in Figure 2C were generated when the remaining strains of *Salmonella* spp. strains as listed in Section “Bacterial Cultures and Genomic DNA Preparation” were subjected to simultaneous AuNPs optical biosensing set at 10 CFU/mL; both blank and non-target changed to purplish blue while no color change was observed on the remaining target samples. Finally, the absorbance (A_625/525nm_) of various *Salmonella* spp. strains reaction mixtures (10 CFU/mL) after adding salt, as shown in Figure 2D, had significant differences at *p* < 0.05 between target and non-target or blank. Based on these results, the AuNPs optical biosensor has successfully and simultaneously detected *Salmonella* spp. strains via *ttrRSBCA* from 10^8^ CFU/mL down to 10 CFU/mL. Based on the color changes differentiation and statistically valid results, the AuNPs optical biosensor has achieved 100% specificity with a DL of <10 CFU/mL. These results were also in agreement with the gel electrophoresis and TEM images (Supplementary Figure S3). DNA concentration measurements of *Salmonella* spp. strains and STEC O157:H7 (non-target) is presented in Supplementary Table S1.

Taxonomically, all 19 *Salmonella* spp. strains that were used in this study belonged to *S. enterica* species and were also environmental and/or outbreak strains with the exception of *S*. Typhimurium ATCC 14028. Twelve of these strains are high on the top 20 list of the most prevalent pathogenic *Salmonella* serotypes in humans in the United States (CDC, 2014). *Salmonella* spp. utilizes tetrathionate as a terminal respiratory electron acceptor and the locus *ttrRSBCA* which is needed for tetrathionate respiration is located near the SPI-2 (Hensel et al., 1999; González-Escalona et al., 2012). SPIs in congruent with other virulence factors, facilitate host cell invasions and intracellular pathogenesis (Marcus et al., 2000). SPIs have been studied so far and each SPI influences the phenotypic characteristics of known *Salmonella* serotypes (Marcus et al., 2000; Morgan, 2007). SPI-2 has two main elements; 25 kb size portion which is only found in *S. enterica* and a 15 kb portion present in both *S. enterica* and *S. bongori* (Marcus et al., 2000; Hensel, 2004). The enzyme tetrathionate reductase is encoded by the structural genes, *ttrA, ttrB* and *ttrC* (Hensel et al., 1999). The 192-bp target region that was identified in this study and has high complementarity with the AuNPs-probes is located on the *ttrA* gene (3,063 bp). Since tetrathionate respiration is an important life cycle event of *Salmonella* spp. strains, these three structural genes are genetically stable which made *ttrRSBCA* an excellent choice as a marker for *Salmonella* spp. diagnostic detection. Based on our extensive literature review, there have been no optical biosensing assay reports that have simultaneously screened and detected *Salmonella* spp. as comprehensive as this study (i.e., nineteen different serovars; Ahn and Walt, 2005; Mazumdar et al., 2007; Valadez et al., 2009; Jokerst et al., 2012; Ohk and Bhunia, 2013; Yang et al., 2013; Viter et al., 2017). Additionally, Table 2 presents previously reported *Salmonella* spp. detection methods and detection limits achieved for selected serovars. Specifically, in comparison to the present study, these methods had longer enrichment periods and/or were only designed for single *Salmonella* spp. strain detection with inferior detection limits. Additionally, since *Salmonella* spp. strains are highly associated with poultry products that often come in a large volume of samples for testing, efficient processing methods and analysis that can save both time and cost without diminishing the sensitivity of the assay are necessary.

**Table 2:** Reported *Salmonella* spp. detection methods (alternative to culture methods) and detection limits achieved for selected serovars.

Detection platform	Method/format	Target foodborne pathogen	Sample matrix	Assay time	Detection limit	Reference
Colorimetric	Nucleic acid-based, gold nanoparticles	*S.* Agona, *S.* Anatum, *S.* Berta, *S.* Derby, *S.* Dublin, *S.* Enteriditis, *S.* Gallinarum, *S.* Heidelberg, *S.* Infantis, *S.* Javiana, *S.* Kentucy, *S.* Mbandaka, *S.* Montevideo, *S.* Muenster, *S.* Newport, *S.* Oranienburg, *S.* Saintpaul, *S.* Senftenberg, *S.* Thompson, *S.* Typhimurium	Blueberry, chicken meat with skin, soil	optical detection ≤ 1 h; enrichment + detection for more than 50 samples < 9 h	<10 CFU/mL or g	This study
Digital PCR	Nucleic acid-based	*S*. Typhimurium	Milk	4 h	10^2^ CFU/mL	Wang et al., 2018
Multiplex loop-mediated isothermal amplification	Nucleic acid-based	*S*. Typhimurium	Milk	20 h	5 CFU/10 mL	Shao et al., 2011
Two-step real-time PCR	Nucleic acid-based	*S*. Typhimurium, *S*. Enteritidis, *S*. Newport, *S*. Heidelberg, and *S*. Hadar	Ground beef	4 h, > 24 h with enrichment	0.156 CFU/g	Bugarel et al., 2017
Multiplex PCR	Nucleic acid-based	*S*. Typhimurium, *S*. Enteritidis, *S*. Typhi	Poultry meat broth	18 h	–	de Freitas et al., 2010
Electrochemical immunosensor	ELISA-based	*S*. Typhimurium	Chicken breast	>24 h	10^2^–10^3^ CFU/mL	Salam and Tothill, 2009
Optical immunochromatographic strip	ELISA-based	*S*. Typhimurium	Water	20 min	10^3^ CFU/mL	Park et al., 2010
Optical	ELISA-aptamer-gold nanoparticles	*S*. Typhimurium	Milk	3 h	10^3^ CFU/mL	Wu et al., 2014
Differential pulse voltammetry	Magneto immuno-based	*S*. Typhimurium LT2	Skimmed milk	1.5 h	143 cells/mL	Afonso et al., 2013
Optical grating coupler (OGC) sensor	Immuno-based	*S*. Typhimurium	Pure culture	–	1.3 × 10^3^ CFU/mL	Kim et al., 2007
Multiplex fiber optic biosensor	Immuno-based	*S*. Enteritidis	Meat (beef, turkey, chicken)	18 h with enrichment	10^3^ CFU/mL	Ohk and Bhunia, 2013
Time-resolved fluorescence (TRF) assay	Immuno-based	*S*. Enteritidis	Egg and chicken breast	2–6 h with enrichment	10^2^ CFU/mL	Valadez et al., 2009
Quartz crystal microbalance (QCM)	Immuno-based	*S*. Typhimurium	Chicken meat	>16 h with enrichment	10–20 CFU/mL	Uludag and Köktürk, 2015
Colorimetric	Immune and gold nanoparticles-based	*S*. Typhimurium	Water	15 min	10^4^ CFU/mL	Wang et al., 2010
Electrochemical aptasensor	Aptamer-based	*S*. Typhimurium	Apple juice	30 min	10^2^ CFU/mL	Bagheryan et al., 2016
Magnetoelastic biosensors	Phage-based	*S*. Typhimurium	Fresh tomato surfaces	30 min	5 × 10^2^ CFU/mL	Li et al., 2010

### Application of the AuNPs Optical Biosensing in Food and Environmental Samples

We designed a pooling technique that allowed processing of a large number of samples (>50 samples) in a short period of time (<9 h) including both enrichment and detection of *Salmonella* spp. strains. Pooled *ttrRSBCA*-negative samples would mean that all the original individual subsamples were not contaminated with *Salmonella* spp. strains. However, individual subsamples were further tested when its original pooled sample source was positive for *ttrRSBCA*. After the short enrichment and sample preparation, the actual simultaneous AuNPs optical biosensing only took ≤1 h to complete.

**FIGURE 3:**
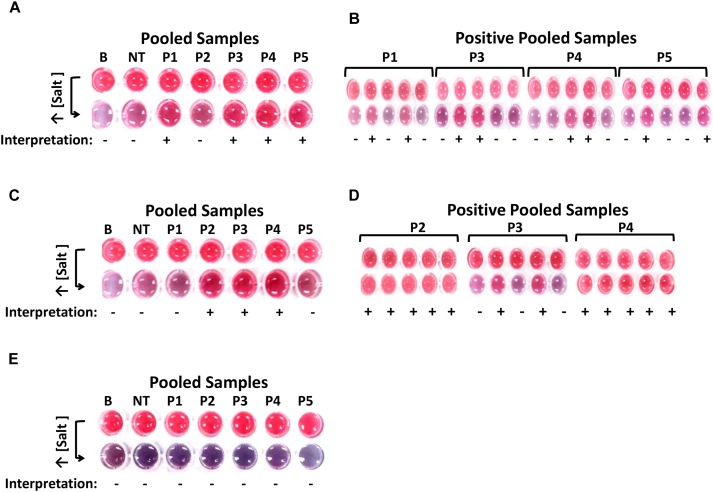
Detection of various *Salmonella* spp. strains in complex matrices. **(A)** After IMS system, five pools (P1-P5) of chicken meat with skin were tested for the presence of *ttrRSBCA*. Among these five pools, P2 tested negative so as its five subsamples. The other 4 pools were positive: P1, P3, P4 and P5. **(B)** From the four positive pools, a total of 8 individual subsamples tested positive for *ttrRSBCA*. **(C)** Three out of 5 blueberry pools: P2, P3 and P4 were positive for *ttrRSBCA.* **(D)** Individual subsamples from the three positive blueberry pools were tested and 12 out of 15 these individual subsamples showed positive for *ttrRSBCA.* **(E)** All five soil sample pools: P1-P5 were negative for *ttrRSBCA.* B = Blank, nuclease-free water; NT = non-target, STEC O157:H7 ATCC 35150.

In Figure 3A, results show that four out of five pooled enriched chicken meat with skin samples tested positive and subsequently in Figure 3B, eight of its 20 subsamples also tested positive for *ttrRSBCA*. For the blueberry setup in Figure 3C, 3 out of five pooled samples were positive and Figure 3D shows that 12 of its 15 subsamples were also positive for *ttrRSBCA*. For the natural and uninoculated environmental soil samples, Figure 3E shows that all samples tested negative for *ttrRSBCA*. A standard plating technique as recommended in the Bacteriological Analytical Manual (BAM) of the US FDA was performed in parallel with the newly developed optical biosensor. In this validation step, HE Agar was used and the results showed development of green colonies with black centers (presumptive positive) for all *ttrRSBCA* positive samples while yellow to salmon orange colonies (presumptive negative) for all *ttrRSBCA* negative samples were observed (Supplementary Figure S4).

In this study, the AuNPs optical biosensor shows efficient simultaneous detection of *Salmonella* spp. When addressing the detection of viable cells from complex matrices, which is an important aspect in the detection of foodborne pathogens in food and environmental samples, the AuNPs optical biosensing coupled with a shortened enrichment and strategic pooling with IMS could process and screen more than 50 samples in <9 h, as compared to the conventional method (>24 h). Sample pooling increases the efficiency of processing large volume of materials that need to be tested (Singer et al., 2006). By collecting naturally pooled fecal-contaminated materials such as from large number of birds, the actual chance of including fecal samples from infected chickens is increased as compared to collection of individual swabs or droppings (Carrique-Mas and Davies, 2008). However, pooling of poultry manures for *Salmonella* spp. detection has a critical dependency on the “dilution effect” in which mixing of negative and positive samples decreases the likelihood of detection (Arnold et al., 2011). Thus, IMS system was incorporated to eliminate the negative impacts of the dilution effect on the sensitivity of the *Salmonella* spp. detection that might arise during the development of the technology. Even if the dilution effects occurred while processing the samples, it would be very negligible and pooling of samples would still mean more efficient as compared to individual sampling for *Salmonell*a spp. detection (Arnold and Cook, 2009). IMS is an efficient tool that separates and concentrates target bacteria from food and environmental samples. This separation of bacteria allows the exclusion of interfering substances that may contribute to non-specificity and cross-reactivity of the downstream assays. Physical separation, capture and concentration of target bacteria add more layers of selectivity from the competitive microbiota especially when dealing with complex matrices.

There have been increasing efforts toward developing and improving rapid detection of *Salmonella* spp. in poultry and poultry products. *Salmonella* outbreaks in the recent years encourage the need for reliable, cost-effective and high-throughput detection methods (Park et al., 2014). A multiplex PCR for the detection of *Salmonella* targeting STM4492 gene and the differentiation of *S*. Typhimurium and *S*. Enteriditis in chicken meat was previously reported with a DL of 10^5^ CFU/g (Saeki et al., 2013). The specificity of the detection method in the present study is based on the highly conserved *ttrRSBCA* region that is present in *Salmonella* spp. strains, its complementary AuNPs-probes as well as the optical features of AuNPs. There was no non-specificity that was observed in this experiment even when the assay was applied in complex matrices, however, the color intensity of the reaction mixtures can still be optimized by further reducing the background noise to provide better resolution for ease of differentiation between target and non-target samples. Future studies may include further quantitative aspects to correlate color intensity with the actual viable cell counts, though additional verification steps may be needed aside from direct observation using the naked eye.

## Conclusion

We have developed a sensitive optical detection platform coupled with an efficient sample pooling in conjunction with an IMS that allowed simultaneous optical biosensing of various viable *Salmonella* spp. strains on contaminated poultry, produce and natural environmental samples. AuNPs were synthesized and surface functionalized with short oligonucleotides that rendered high stability without compromising its biocompatibility. Sandwich hybridization between specific AuNPs-probes and highly conserved regions of *ttrRSBCA* locus provided the opportunity to optically differentiate target and non-target samples after increasing the salt concentration of the reaction mixtures. The results showed that the highly sensitive assay toward its target with a superior DL of <10 CFU/mL or g and 100% specificity. The total turn-around time with enrichment is <9 h for detection of viable *Salmonella* spp. in more than 50 food and environmental samples tested. This approach has significantly shortened the screening process for simultaneously detecting outbreak and environmental strains of *Salmonella* spp. as compared to the conventional method. The unique colorimetric properties of AuNPs have provided this assay a simple and stable platform at a very low cost, allowing rapid screening of a highly significant group of foodborne pathogens.

## Data Availability

All datasets generated for this study are included in the manuscript and/or the Supplementary Files.

## Author Contributions

VW and IQ designed the experiments and performed data analysis. IQ conducted the experiments including TEM imaging. IQ prepared the manuscript, VW, C-SL, and BdlR edited the manuscript. All authors read and approved the final manuscript.

## Conflict of Interest Statement

The authors declare that the research was conducted in the absence of any commercial or financial relationships that could be construed as a potential conflict of interest.
