# Supplementary material for: Simultaneous Colorimetric Detection of a Variety of Salmonella spp. in Food and Environmental Samples by Optical Biosensing Using Oligonucleotide-Gold Nanoparticles
Source: Front Microbiol. 2019 May 31;10:1138. doi: 10.3389/fmicb.2019.01138 (PMC6554661; doi:10.3389/fmicb.2019.01138)
Supplement: Supplementary file 1 [file Data_Sheet_1.docx]

Supplementary Material

***ttrRSBCA***

**5’**- ACCCACGCGT…**CGTCAAGACCGTTTCTCCCGCCAGTTGCT**-CC…..AAA-**GGGACGGACGAGTCAATCGGGTGTTCCTGC**…TGCCGGC - **3’**

**AuNP**-SH-(CH_2_)_6_- **GCAGTTCTGGCAAAGAGGGCGGTCAACGA** - **5’**  **3’** - **CCCTGCCTGCTCAGTTAGCCCACAAGGACG** -(CH_2_)_6_ -SH-**AuNP**

**_Probe 1 Probe 2_**

**Figure S1**. Thiolated Probes 1 and 2 (30-mer each) immobilized on the surface on AuNPs hybridizing with its complementary strands. The conserved region (192-bp) of *ttrRSBCA* locus was the target site for annealing for sandwich hybridization. 5’- or 3’- end of the probes had thiol linkage, HS-(CH_2_)_6_ for AuNPs conjugation.


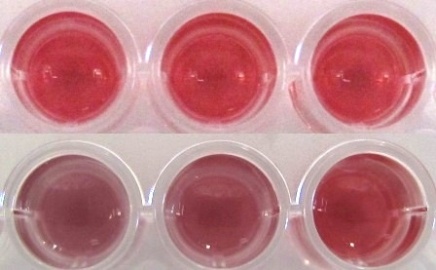


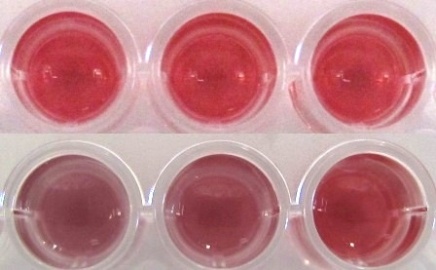


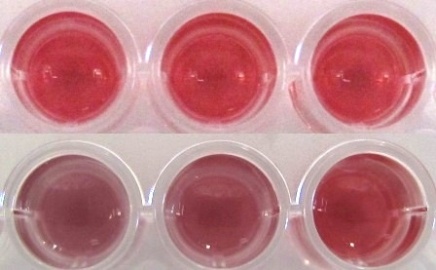


**Figure S2**. Immobilization of probes onto the surface of AuNPs. Functionalization of AuNPs involved immobilization of thiolated oligonucleotides can cause a shift (2-3 nm) in the absorbance peak of AuNPs.

**(A)**

**7 – *S*. Heidelberg**

**9 – *S*. Javiana**

**6 – *S*. Gallinarum**

**8 – *S*. Infantis**

**5 – *S*. Enteridtis**

**3 – *S*. Derby**

**1 – *S*. Anatum**

**4 – *S*. Dublin**

**2 – *S*. Berta**

**O157:H7**

**Blank**


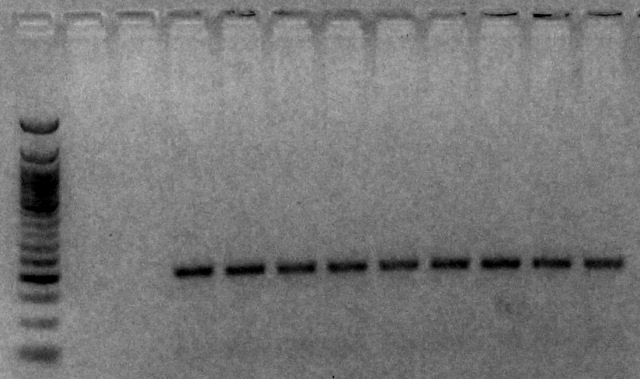


**200 bp**

**14 – *S*. Newport**

**16 – *S*. Saintpaul**

**18 – *S*. Thompson**

**19 – *S*. Typhimurium**

**17 – *S*. Senftenberg**

**15 – *S*. Oranienburg**

**13 – *S*. Muenster**

**12 – *S*. Montevideo**

**10 - *S*. Kentucky**

**11- *S*. Mbandaka**


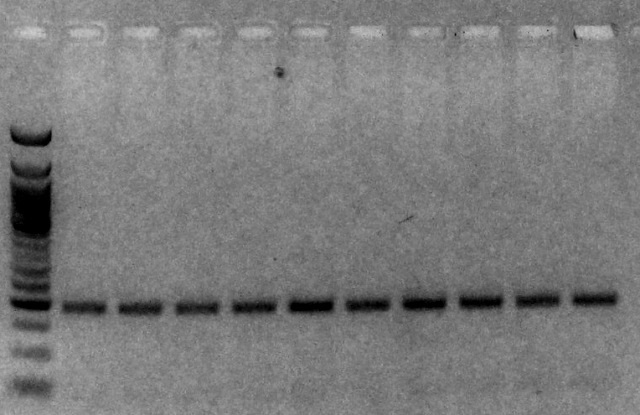


**200 bp**

**(B)**


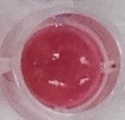


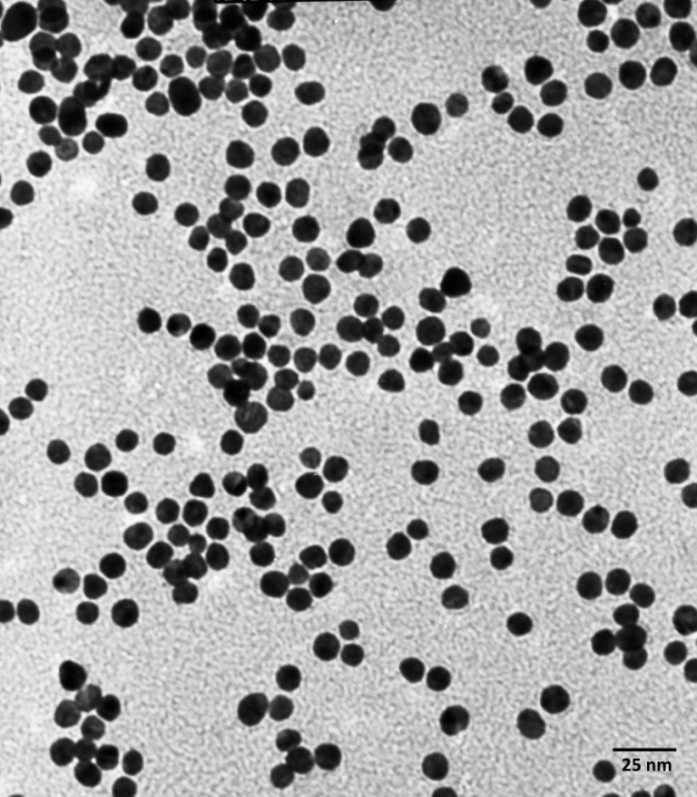
 **
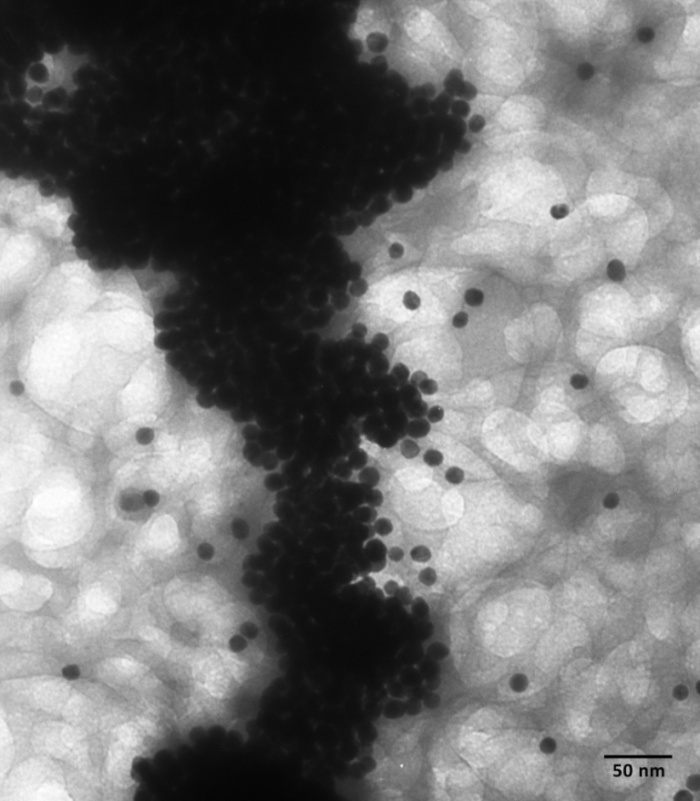
**


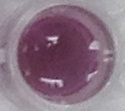


**(C)**


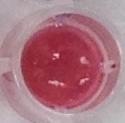


**
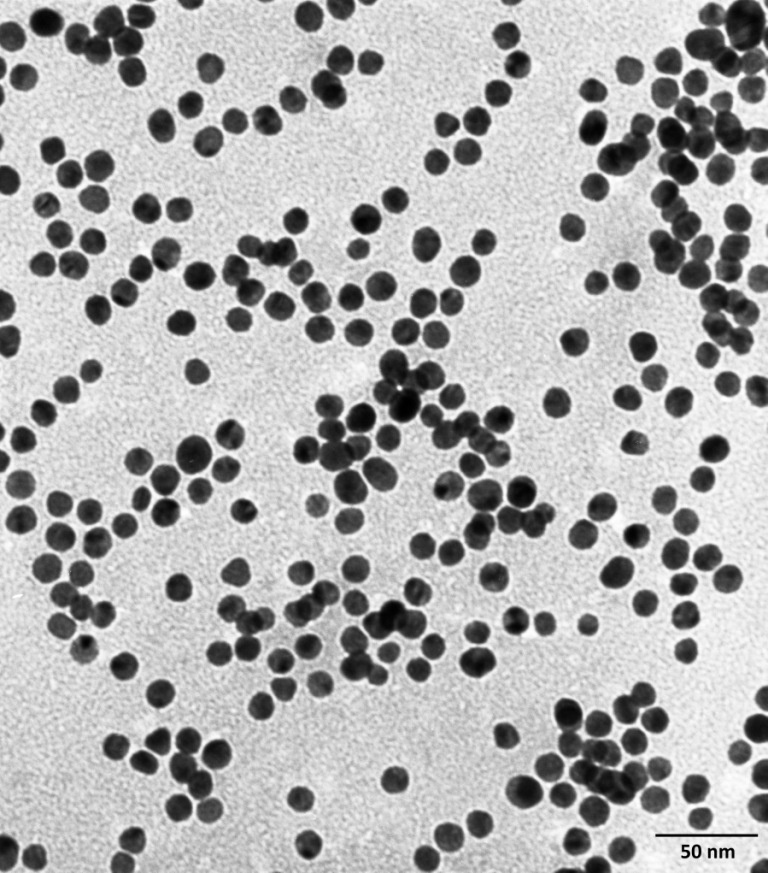

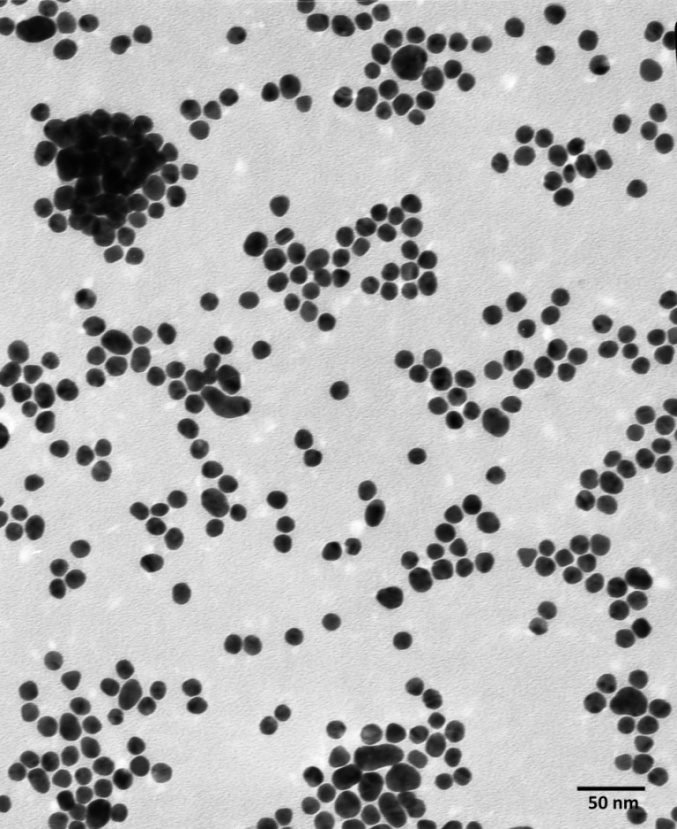
**


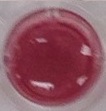


**Figure S3.** Confirmation of oligonucleotide-AuNPs-DNA sandwich hybridization and color challenge test. (A) Gel electrophoresis of reaction mixtures after sandwich hybridization showing the 192-bp bands from target *Salmonella* spp. samples. (B) TEM images of non-target samples before (left micrograph) and after (right micrograph) adding salt solution. Intense aggregation can be seen due to high salt concentration and absence of oligonucleotide-AuNPs-DNA complexes. (C) TEM images of target samples before (left micrograph) and after (right micrograph) adding salt solution. Uniform dispersion of AuNPs can be seen due to the presence of oligonucleotide-AuNPs-DNA complexes even after increased salt concentration.

**Table S1**. DNA concentration measurements of *Salmonella* spp. strains and STEC O157:H7 (non-target) after asPCR. Initial concentration of pure *Salmonella* spp. and STEC O157:H7 strains cultures was 1 log CFU/ml prior to DNA extraction. Data (ng/µl) are expressed as mean ± standard deviation (n=3)). Nanodrop 2000 (Fisher) was used for measurement.

| **Target DNA** | **Source** | **DNA Concentration** |
| --- | --- | --- |
| *ttrRSBCA* from 1 log CFU/ml cultures | *S.* Agona | 340.5 ± 24.47 |
|  | *S.* Anatum | 364.65 ± 23.97 |
|  | *S.* Berta | 319.15 ± 0.35 |
|  | *S.* Dublin | 330.15 ± 17.89 |
|  | *S.* Enteriditis | 330.95 ± 13.36 |
|  | *S.* Gallinarum | 324.55 ± 1.63 |
|  | *S.* Heidelberg | 331.05 ± 17.61 |
|  | *S.* Infantis | 368.1 ± 77.92 |
|  | *S.* Javiana | 369.55 ± 80.54 |
|  | *S.* Mbandaka | 359.2 ± 95.74 |
|  | *S.* Montevideo | 361.65 ± 104.30 |
|  | *S.* Newport | 371.1 ± 90.51 |
|  | *S.* Oranienburg | 349.05 ± 105.43 |
|  | *S.* Saintpaul | 350.5 ± 104.79 |
|  | *S.* Senftenberg | 357.85 ± 94.68 |
|  | *S*. Thompson | 373.45 ± 84.22 |
|  | *S*. Derby | 470.45 ± 214.18 |
|  | *S*. Kentucky | 361 ± 85.70 |
|  | *S*. Muenster | 364 ± 96.31 |
|  | STEC O157:H7 | 176.4 ± 17.96 |

**(A)**

**
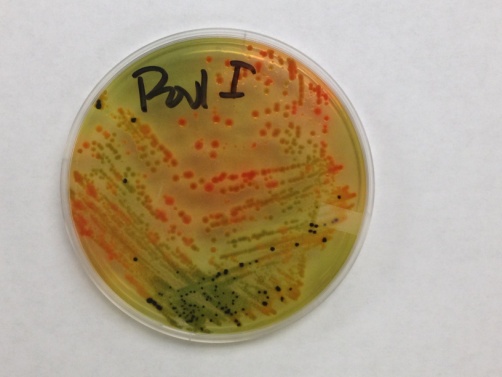

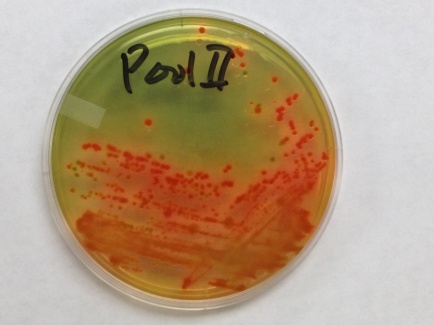

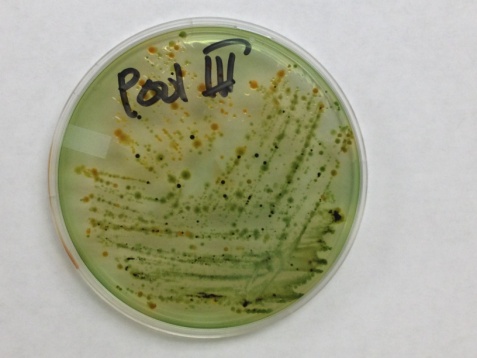
**

Pool 1 (+) Pool 2 (-) Pool 3 (+)

**
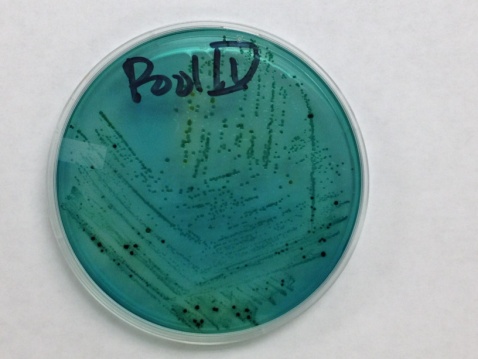

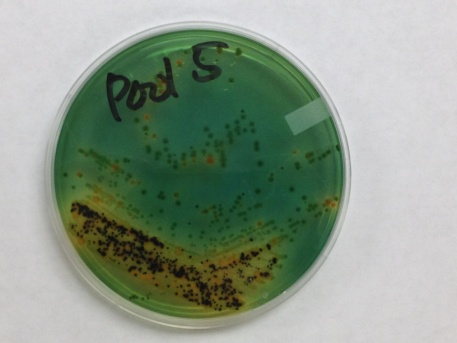
**

Pool 4 (+) Pool 5 (+)

**(B)**

**
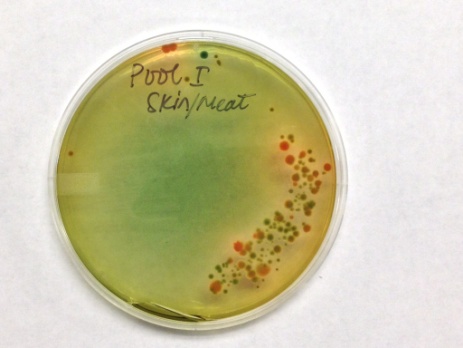

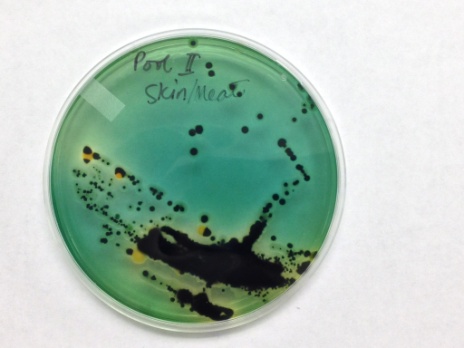

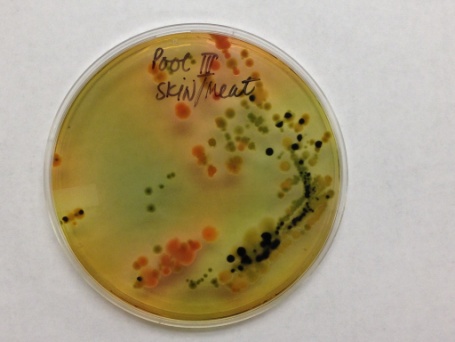
**

Pool 1 (-) Pool 2 (-) Pool 3 (+)

**
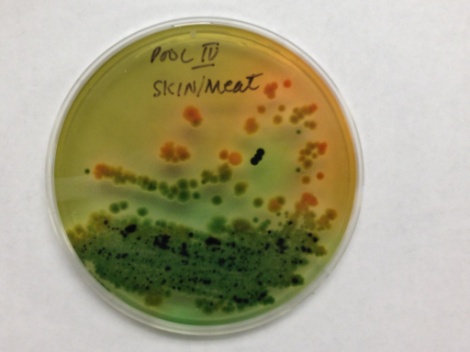

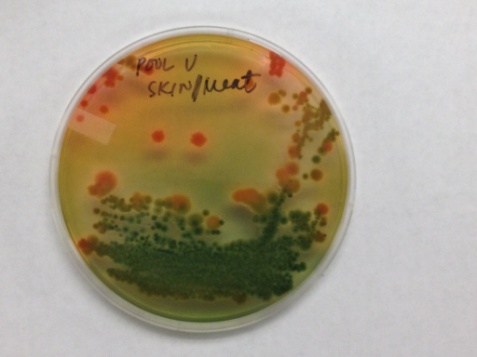
**

Pool 4 (+) Pool 5 (-)

**Figure S4.** Plating of pools using selective medium, HEA. **(A)** Blueberry pools on HEA showing four pools of presumptive positive colonies for *Salmonella* spp. **(B)** Chicken meat on HEA showing three pools of presumptive positive colonies for *Salmonella* spp.
